# The Efficacy and Safety of Jaungo, a Traditional Medicinal Ointment, in Preventing Radiation Dermatitis in Patients with Breast Cancer: A Prospective, Single-Blinded, Randomized Pilot Study

**DOI:** 10.1155/2016/9481413

**Published:** 2016-03-15

**Authors:** Moonkyoo Kong, Deok-Sang Hwang, Jee Young Lee, Seong Woo Yoon

**Affiliations:** ^1^Department of Radiation Oncology, Kyung Hee University Medical Center, Kyung Hee University School of Medicine, 23 Kyungheedae-gil, Dongdaemun-gu, Seoul 130-702, Republic of Korea; ^2^Department of Korean Medicine Obstetrics & Gynecology, Kyung Hee University Medical Center, College of Korean Medicine, Kyung Hee University, 23 Kyungheedae-gil, Dongdaemun-gu, Seoul 130-702, Republic of Korea; ^3^Department of Korean Internal Medicine, Korean Medicine Cancer Center, Kyung Hee University Hospital at Gangdong, College of Korean Medicine, Kyung Hee University, 23 Kyungheedae-gil, Dongdaemun-gu, Seoul 130-702, Republic of Korea

## Abstract

*Purpose*. This study was performed to evaluate the efficacy and safety of Jaungo in preventing radiation dermatitis in patients with breast cancer.* Methods*. Thirty patients were prospectively enrolled and randomly assigned to receive Jaungo or general supportive skin care. Radiation dermatitis and pain were examined at daily intervals from the start of radiotherapy until 4 weeks after its completion. The primary endpoint of this study was the incidence of radiation dermatitis. The secondary endpoints were time to onset of radiation dermatitis, duration of radiation dermatitis, and maximum pain score.* Results*. Jaungo reduced the incidence of grade ≥2 (46.7% versus 78.6%) and grade 3 radiation dermatitis (20.0% versus 50.0%) in comparison with general supportive skin care. Jaungo also delayed the onset of grade 2 dermatitis (35 days versus 30 days). In terms of time to onset of grade 3 dermatitis, duration of dermatitis, and maximum pain score, Jaungo showed results comparable to those achieved with general supportive skin care. No patients experienced adverse effects caused by Jaungo administration.* Conclusions*. Jaungo minimized radiation dermatitis in patients with breast cancer without causing adverse effects. Further randomized studies with a larger sample size are required to assess clinical use of Jaungo.

## 1. Introduction

Radiation dermatitis is the most common complication of radiotherapy (RT) and can impair the quality of life of patients with breast cancer as a result of pain associated with the condition [1, 2]. Several clinical studies have been conducted to evaluate the efficacy of topical agents in preventing and treating radiation dermatitis; however, sufficient evidence to support the efficacy of any particular agent has not been found [3, 4]. Consequently, no clear guidelines have been established for preventing and treating radiation dermatitis in patients with breast cancer.

In recent years, the interest of patients and physicians in using natural products to treat modern medical conditions has increased. Many natural products claim to have beneficial effects, but most do not have sufficient scientific evidence to support their claims [5, 6]. In Korea, Jaungo (Shiunko), a traditional medicinal ointment, is commonly used for treating skin wounds such as cuts, abrasions, frostbite, and burns. Jaungo is composed of* Lithospermi radix* and* Angelica sinensis*.* Lithospermi radix* contains shikonin, acetylshikonin, dimethyacrylshikonin, deoxyshikonin, and isobutylshikonin.* Angelica sinensis* consists of ferulic acid and decursin. Recent studies have demonstrated the antibacterial and anti-inflammatory effects of Jaungo, as well as its capacity to promote wound healing by reepithelization and angiogenesis [7, 8]. In addition, recent studies reported that Jaungo showed favorable effects on radiation-induced scalp dermatitis in patients with brain tumors [9, 10]. This study was performed to evaluate the efficacy and safety of Jaungo in preventing radiation dermatitis in patients with breast cancer.

## 2. Materials and Methods

Patient eligibility criteria included the presence of pathologically confirmed unilateral breast cancer with no tumor invasion of the skin, completion of breast conserving surgery with or without adjuvant chemotherapy, receipt of postoperative RT to the whole breast without bolus, and planned RT dose of ≥45 Gy. Patients with inflammatory breast cancer, an unhealed wound in the breast, a history of prior RT to the chest wall, or a history of connective tissue disorders were excluded. Patients who received concurrent chemotherapy were also excluded. Treatment of regional lymph nodes and concurrent hormone therapy were permitted. The Institutional Review Board of our institution approved this study. All research was carried out in compliance with the Helsinki Declaration (KMC IRB 1415-01). Informed consent was obtained from all patients. Because this is a pilot study, it was not registered with the Clinical Research Information Service.

From December 2014 through July 2015, 30 patients were prospectively enrolled in this study and randomly assigned to receive Jaungo (intervention group) or general supportive skin care (control group). The randomization was stratified according to adjuvant chemotherapy status (yes versus no) using a computer-generated random number list. The patients assigned to the intervention group were instructed to apply Jaungo (Jaungo®, Hanpoong Pharm & Foods Corporation, Seoul, Republic of Korea) on their irradiated skin twice daily, shortly after RT and in the evening. To decrease the possibility of any bolus effect, Jaungo was not applied within 4 hours of the daily RT session. In addition, patients were instructed to clean the ointment from the irradiated area gently with water and a soft towel before the daily RT session. Treatment was started at the onset of RT and continued until 2 weeks after RT was completed or until radiation dermatitis subsided. Treatment was ceased when a patient showed an adverse reaction to Jaungo. Jaungo mainly consists of* Lithospermi radix*,* Angelica sinensis*, sesame oil, and beeswax. The primary bioactive constituents of Jaungo are shikonin (0.07 mg/g) and decursin (3.6 mg/g). Jaungo is manufactured and regulated according to the guidelines of the Korea Food & Drug Administration. The patients assigned to the control group were instructed to keep the irradiated skin clean and dry by gentle washing with neutral pH soap and patting with a soft towel. No prophylactic creams or lotions for radiation dermatitis were allowed to either group. Compliance with the instructions for applying the study cream and general supportive skin care was evaluated weekly by one of the investigators.

All patients received computed tomography-planned RT with the forward field-in-field technique. RT was delivered using a photon beam to the whole breast. With a schedule of 2 Gy per fraction and five fractions weekly, the whole breast was treated with tangential fields to 46–50 Gy. Regional lymph nodal irradiation was delivered to patients with risk factors for regional recurrence (lymphovascular invasion, positive axillary lymph nodes, or extranodal extension) with a total dose of 50–60 Gy. In patients with risk factors for local recurrence (close resection margin or high-grade tumor), an electron boost to the tumor bed with an additional dose of 9–15 Gy was implemented (3 Gy per fraction and five fractions weekly). A bolus was not used in any of the patients.

Radiation dermatitis and pain were examined at daily intervals from the start of RT until 4 weeks after its completion. To minimize interscorer variability, only one investigator, who was blinded to patient assignment, scored the grade of radiation dermatitis according to Radiation Therapy Oncology Group guidelines. The patients' evaluation of pain within the RT field was assessed with a 10 cm visual analog scale. Any adverse events associated with Jaungo treatment were assessed using the Common Terminology Criteria for Adverse Events, version 4.0. At the end of the study, patients in the intervention group were asked to fill out a simple questionnaire to assess their preference for the ointment regarding smell, color, and ease of application. During treatment, RT interruptions due to severe radiation dermatitis were also documented.

The primary endpoint of this study was the incidence of radiation dermatitis. The secondary endpoints were time to onset of radiation dermatitis, duration of radiation dermatitis, and maximum pain score. Baseline characteristics between groups were compared using a chi-square test for discrete variables and an independent *t*-test for continuous variables. To assess differences in the incidence of radiation dermatitis between the two groups, we compared actuarial rates estimated using the Kaplan-Meier method, whereas comparisons between groups were performed using a log-rank test. Elapsed time was calculated from the date of initiation of RT to the date of occurrence of events or final follow-up visit. Time to onset of radiation dermatitis, duration of radiation dermatitis, and maximum pain score were compared using the Mann-Whitney *U* test. The duration of radiation dermatitis was calculated as the number of days from the onset of radiation dermatitis to the date when it was resolved. Patients who did not experience radiation dermatitis were assigned a duration of 0 days. All analyses were performed using SPSS version 18.0 (IBM Corporation, Armonk, NY, USA). All tests were two-sided and *P* < 0.05 was considered statistically significant.

## 3. Results

**Figure 1:**
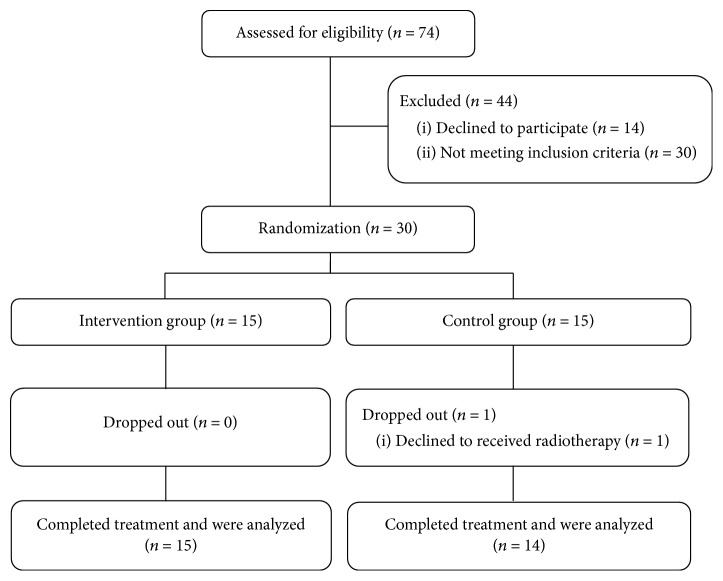
Flow chart of patients in Jaungo pilot study.

Twenty-nine of 30 patients completed the planned RT and complied well with instructions for applying the study cream and general supportive skin care. One patient in the control group refused to receive RT after undergoing 5 sessions of RT. Therefore, 15 patients in the intervention group and 14 patients in the control group were included in the efficacy and safety analyses (Figure 1). Patient characteristics are summarized in Table 1. There were no significant differences in patient or tumor characteristics between the two groups. 9 patients in the intervention group and 10 patients in the control group received tumor bed boost RT. All patients had never smoked and were followed up until 4 weeks after completion of RT.

**Figure 2:**
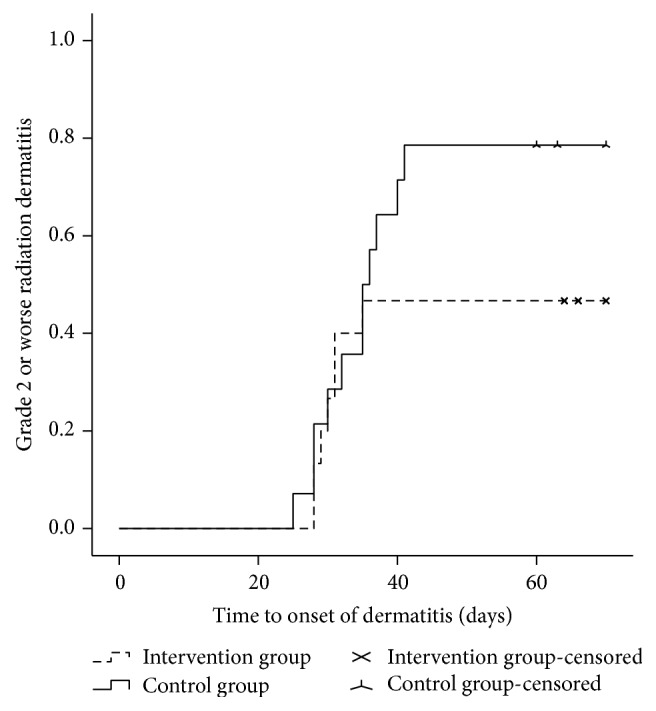
Incidence of grade ≥2 radiation dermatitis in patients who received Jaungo or general supportive skin care. Patients who applied Jaungo on their irradiated skin had a lower incidence of grade ≥2 radiation dermatitis compared to patients who received general supportive skin care (46.7% versus 78.6%).

**Figure 3:**
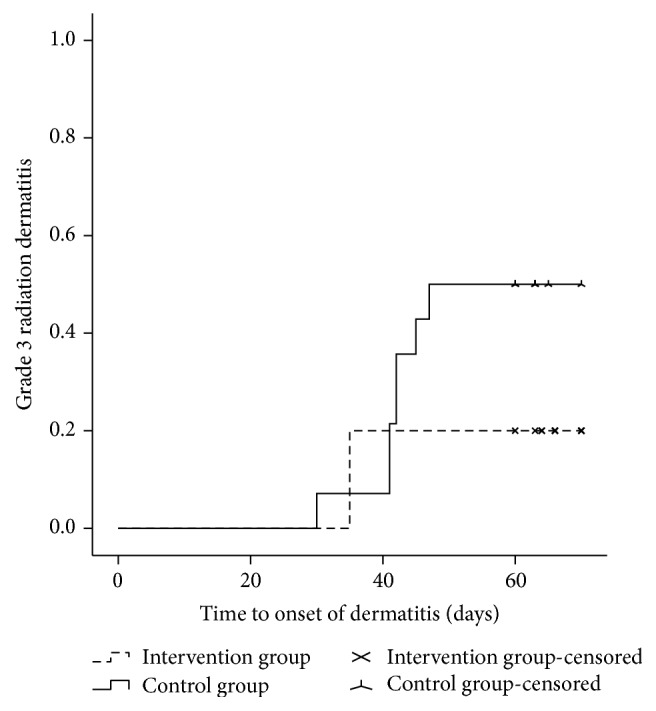
Incidence of grade 3 radiation dermatitis in patients who received Jaungo or general supportive skin care. Patients who applied Jaungo on their irradiated skin had a lower incidence of grade 3 radiation dermatitis compared to patients who received general supportive skin care (20.0% versus 50.0%).

The results for the primary and secondary endpoints are summarized in Table 2. In the whole evaluable patient population, maximum grade 2 and grade 3 radiation dermatitis developed in 18 (62.1%) and 10 patients (34.5%), respectively. All patients experienced grade ≥1 radiation dermatitis, while no patient experienced grade 4 dermatitis. One patient in the control group experienced an RT interruption for 10 days because of a severe skin reaction. Grade ≥2 radiation dermatitis developed in 7 (46.7%) and 11 patients (78.6%) in the intervention group and control group, respectively (*P* = 0.095) (Figure 2). Grade 3 dermatitis developed in 3 (20.0%) and 7 patients (50.0%) in the intervention group and control group, respectively (*P* = 0.093) (Figure 3). The use of Jaungo reduced the incidence of grade ≥2 and grade 3 radiation dermatitis; however, these differences were not statistically significant.

In the whole evaluable patient population, the median time to onset of grade 2 and grade 3 radiation dermatitis was 31 (range, 25–41 days) and 41 days (range, 30–47 days), respectively. The median time to onset of grade 2 dermatitis was 35 days (range, 25–41 days) in the intervention group and 30 days (range, 28–35 days) in the control group (*P* = 0.164). Jaungo delayed the onset of grade 2 dermatitis, but its effect was not statistically significant. However, in terms of grade 3 dermatitis, the use of Jaungo showed conflicting results. In the intervention group, all grade 3 dermatitis developed at 38 days after initiation of RT. In comparison, in the control group, the median time to onset of grade 3 dermatitis was 42 days (range, 30–47 days) (*P* = 0.117).

In the analysis of the duration of radiation dermatitis, Jaungo showed results comparable to those achieved with general supportive skin care. The median duration of grade ≥2 dermatitis was 15 days (range, 7–37 days) in the intervention group and 16 days (range, 14–36 days) in the control group (*P* = 0.479). The median duration of grade 3 dermatitis was 10 days (range, 9–11 days) in the intervention group and 11 days (range, 8–25) in the control group (*P* = 0.517).

In the whole evaluable patient population, the median maximum pain score was 4.1 (range, 1–8.5). The median maximum pain score was 4 (range, 1–8) in the intervention group and 4.5 (range, 3–8.5) in the control group (*P* = 0.158). No patients experienced adverse effects caused by Jaungo administration. All patients in the intervention group completed a simple questionnaire to assess their preference for Jaungo. Two patients were repulsed by the deep red color of Jaungo.

## 4. Discussion

Although many agents are used clinically to prevent and treat radiation dermatitis [5, 6, 11–16], several recent systematic reviews have reported the absence of sufficient evidence to support the use of any particular agent in preventing or treating radiation dermatitis in patients with breast cancer; therefore, new agents with therapeutic potential in patients with radiation dermatitis associated with breast cancer treatment are of significant interest [3, 4, 17]. Natural products with anti-inflammatory and/or wound healing properties could prevent or minimize radiation dermatitis and thus serve as alternatives to synthetic compounds. Jaungo is composed of natural products that have been reported to possess anti-inflammatory and wound healing properties.* Angelica sinensis* is a basic component of many traditional herbal drugs used to accelerate wound healing. Ferulic acid, the primary bioactive constituent of* Angelica sinensis*, has been shown to promote wound healing in a diabetic rat model [7, 18].* Lithospermi radix* is commonly used to treat skin wounds such as cuts, abrasions, frostbite, and burns. Acetylshikonin, a bioactive constituent of* Lithospermi radix*, promotes wound healing and has an antibacterial effect [8]. Furthermore, recent studies report that Jaungo ointment could be used to prevent radiation-induced scalp dermatitis in patients with brain tumors [9, 10]. Given this background, we evaluated the efficacy and safety of Jaungo in preventing radiation dermatitis in patients with breast cancer.

In our study, no patient experienced adverse effects caused by Jaungo. Jaungo reduced the incidence of grade ≥2 (46.7% versus 78.6%) and grade 3 radiation dermatitis (20.0% versus 50.0%) in comparison with general supportive skin care. This pilot study was conducted to investigate whether Jaungo is safe and effective and to compare its effect on the incidence of radiation dermatitis with that of general supportive skin care in a descriptive manner. Statistical significance was not an essential assessment criterion, because the results were expected to form the basis for the power calculation for a further large-scale study. Although we did not find obvious differences in secondary endpoints (time to onset of radiation dermatitis, duration of radiation dermatitis, and maximum pain score), Jaungo seemed to reduce the incidence of radiation dermatitis. Based on the results of this pilot study, we plan to conduct additional studies with larger sample sizes to confirm the efficacy of Jaungo in preventing radiation dermatitis in patients with breast cancer.

The difficulty in determining the efficacy of topical agents may be in part attributable to subjectivity in the scoring criteria for radiation dermatitis. Because most scoring criteria for radiation dermatitis are based on subjective evaluation by investigators, interobserver and/or intraobserver variation may be present. In this study, in order to minimize interobserver and/or intraobserver variation, only one investigator, who was blinded to patient assignment and had more than 10 years of experience in evaluating and treating radiation toxicity, scored the grade of radiation dermatitis. Because patients in the intervention group were instructed not to apply Jaungo within 4 hours of each daily RT session and to clean the ointment from the irradiated skin before starting the daily RT session, the blindness of the investigator who scored the grade of radiation dermatitis was maintained.

In this study, no patients experienced adverse effects caused by Jaungo administration. However, two patients were repulsed by the deep red color of Jaungo. Jaungo has a deep red color because it is composed of* Lithospermi radix*. Although all patients in the intervention group complied well with the instructions for applying Jaungo, a detailed explanation regarding the deep red color of Jaungo might reduce patients' feeling of repulsion toward applying Jaungo in future studies.

This study has some limitations. First, this is a pilot study with a small sample size. Second, because the patients were not blinded to their group assignment, possible confounding factors might have been present. Therefore, the results of this study do not allow us to make definite conclusions regarding the safety and efficacy of Jaungo. However, this is the first study to evaluate the efficacy of Jaungo on radiation dermatitis in patients with breast cancer. Moreover, the two experimental groups were extremely well randomized and comparable according to their similar patient and tumor characteristics. We believe that the evidence provided in this study will encourage further studies exploring the efficacy of Jaungo in preventing radiation dermatitis associated with cancer treatment.

In the present single-blinded, randomized pilot study, Jaungo was found to minimize radiation dermatitis in patients with breast cancer without causing adverse effects. To confirm the results of this study, well-designed randomized studies with a larger sample size are required.

## Figures and Tables

**Table 1:** Patient characteristics.

	Intervention group (*n* = 15)	Control group (*n* = 14)	*P* value
Age (years)			
Median (range)	54.1 (46.4–74.2)	50.7 (40.1–70.8)	0.368
Diabetes mellitus			
Yes/no	1/14	1/13	0.911
ECOG performance status			
0/1	2/13	1/13	0.834
Body mass index (kg/m^2^)			
Median (range)	23.5 (19.9–32.2)	23.2 (16.6–29.7)	0.162
Breast size^†^ (cc)			
Median (range)	364.8 (208.9–609.2)	372.4 (200.8–568.3)	0.345
Site			
Right/left	5/10	5/9	0.874
Histology			
Ductal carcinoma	11	11	0.826
Nonductal carcinoma	4	3	
T stage			
In situ	4	2	0.391
1	6	7	
2	5	5	
N stage			
0	14	13	0.934
1	1	1	
Molecular subtypes			
Luminal	10	12	0.187
Triple negative	2	1	
HER2-positive	3	1	
Total RT dose (Gy)			
Median (range)	55 (46–61)	58 (46–58)	0.571
Lymph node irradiation			
Yes/no	1/14	0/14	0.423
Adjuvant chemotherapy			
Yes/no	6/9	5/9	0.721
Concurrent hormone therapy			
Yes/no	9/6	10/4	0.627
Concurrent herceptin therapy			
Yes/no	1/14	1/13	0.857

^†^Calculated from the clinical target volume (CTV) of the whole breast using the radiotherapy planning computer

ECOG, Eastern Cooperative Oncology Group; HER2, human epidermal growth factor receptor 2; RT, radiotherapy.

**Table 2:** Analyses of primary and secondary endpoints.

Endpoint	Intervention group	Control group	*P* value
Incidence of radiation dermatitis (%)			
Grade ≥ 2	46.7	78.6	0.095
Grade 3	20.0	50.0	0.093
Time to onset of radiation dermatitis (days)			
Grade 2 [median (range)]	35 (25–41)	30 (28–35)	0.164
Grade 3 [median (range)]	38	42 (30–47)	0.117
Duration of radiation dermatitis (days)			
Grade ≥ 2 [median (range)]	15 (7–37)	16 (14–36)	0.479
Grade 3 [median (range)]	10 (9–11)	11 (8–25)	0.517
Pain score [median (range)]	4.0 (1.0–8.0)	4.5 (3.0–8.5)	0.158

RT, radiotherapy.
